# Insights into the phylogenetic relationships and drug targets of *Babesia* isolates infective to small ruminants from the mitochondrial genomes

**DOI:** 10.1186/s13071-020-04250-8

**Published:** 2020-07-29

**Authors:** Xiaoxing Wang, Jinming Wang, Junlong Liu, Aihong Liu, Xin He, Quanjia Xiang, Youquan Li, Hong Yin, Jianxun Luo, Guiquan Guan

**Affiliations:** 1 State Key Laboratory of Veterinary Etiological Biology, Key Laboratory of Veterinary Parasitology of Gansu Province, Lanzhou Veterinary Research Institute, Chinese Academy of Agricultural Science, Xujiaping 1, Lanzhou, 730046 Gansu People’s Republic of China; 2 Jiangsu Co-Innovation Center for the Prevention and Control of Important Animal Infectious Disease and Zoonoses, Yangzhou University, Yangzhou, 225009 People’s Republic of China

**Keywords:** *Babesia motasi*, *Babesia* sp., Cytochrome *bc*1 complex, Drug target, Mitochondrial genome, Phylogenetic relationship

## Abstract

**Background:**

Babesiosis, a tick-borne disease caused by protozoans of the genus *Babesia*, is widespread in subtropical and tropical countries. Mitochondria are essential organelles that are responsible for energy transduction and metabolism, calcium homeostasis and cell signaling. Mitochondrial genomes could provide new insights to help elucidate and investigate the biological features, genetic evolution and classification of the protozoans. Nevertheless, there are limited data on the mitochondrial genomes of ovine *Babesia* spp. in China.

**Methods:**

Herein, we sequenced, assembled and annotated the mitochondrial genomes of six ovine *Babesia* isolates; analyzed the genome size, gene content, genome structure and cytochrome *b* (*cytb*) amino acid sequences and performed comparative mitochondrial genomics and phylogenomic analyses among apicomplexan parasites.

**Results:**

The mitochondrial genomes range from 5767 to 5946 bp in length with a linear form and contain three protein-encoding genes, cytochrome *c* oxidase subunit 1 (*cox*1), cytochrome *c* oxidase subunit 3 (*cox*3) and *cytb*, six large subunit rRNA genes (*LSU*) and two terminal inverted repeats (TIR) on both ends. The *cytb* gene sequence analysis indicated the binding site of anti-*Babesia* drugs that targeted the cytochrome *bc*1 complex. *Babesia microti* and *Babesia rodhaini* have a dual flip-flop inversion of 184–1082 bp, whereas other *Babesia* spp. and *Theileria* spp. have one pair of TIRs, 25–1563 bp. Phylogenetic analysis indicated that the six ovine *Babesia* isolates were divided into two clades, *Babesia* sp. and *Babesia motasi*. *Babesia motasi* isolates were further separated into two small clades (*B. motasi* Hebei/Ningxian and *B. motasi* Tianzhu/Lintan).

**Conclusions:**

The data provided new insights into the taxonomic relationships and drug targets of apicomplexan parasites. 
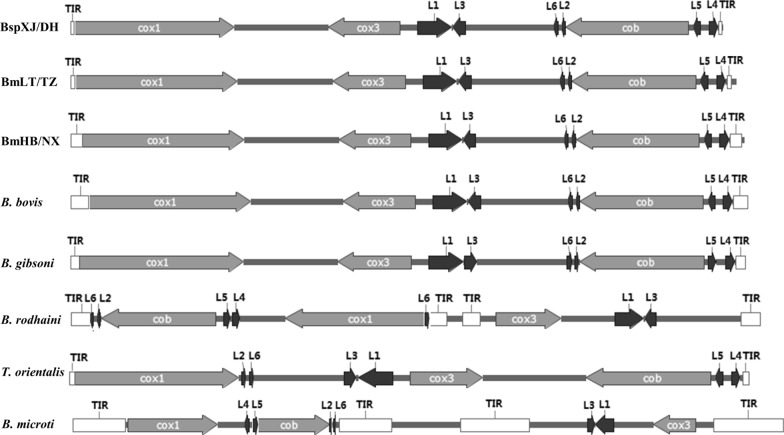

## Background

Babesiae are tick-transmitted hemoprotozoans that cause babesiosis, which is characterized by fever, anemia, jaundice and hemoglobinuria. The main causative agents in small ruminants are *Babesia ovis* and *B. motasi*, transmitted by the ticks *Rhipicephalus* spp. and *Haemaphysalis* spp. in Asia, South America, Africa, the Far East and Europe [1–3]. In China, several *Babesia* isolates were isolated from sheep during the period of 2000–2010, *Babesia* sp. Dunhuang (BspDH), *Babesia* sp. Xinjiang (BspXJ), *B. motasi* Tianzhu (BmTZ), *B. motasi* Lintan (BmLT), *B. motasi* Hebei (BmHB) and *B. motasi* Ningxian (BmNX). The six ovine *Babesia* parasites have different characteristics in serology, pathogenicity, vector specificity and virulence. For instance, BspXJ and BspDH have low virulence and are transmitted by *Hyalomma* spp. ticks, whereas the vector ticks of BmLT, BmHB, BmNX and BmTZ are *Haemaphysalis* spp. and cause a range of clinical manifestations. BspXJ/DH is serologically distinct from *B. motasi*, and there are also differences between isolates of *B. motasi* [4–12]. The prevalences of *B. motasi* and *Babesia* sp. were between 30.4–31.7 and 36.0–43.5%, respectively, which indicates that *Babesia* infection is prevalent in sheep and goats in China [11, 13–15].

Mitochondria are crucial organelles that are responsible for energy transduction and metabolism, calcium homeostasis and cell signaling [16]. The mitochondrial cytochrome *bc*1 complex, known as complex III, is a multimeric enzyme that is an indispensable element of the respiratory chain and energy conversion [17]. Complex III comprises three redox active subunits, cytochrome *b* (encoded by the mitochondrial genome), cytochrome *c* oxidase subunit 1 and the Rieske iron-sulfur protein. The catalytic cycle of cytochrome *bc*1 (Q cycle) is in quinone reduction (Qi) and quinol oxidation (Qo) sites that are mainly form by cytochrome *b*. Inhibitors of the *bc*1 complex may be divided into two types that act on Qi and Qo sites according to the binding site. Therefore, the *bc*1 complex has been considered a promising target for the development of antimicrobial compounds. Some studies suggest that the yeast *bc*1 complex structure could be used as a model for discovering new antimalarial drugs [18, 19]. However, mutations in the cytochrome *b* (*cytb*) gene have been shown to be the molecular basis of drug resistance of some microorganisms [19–22]. It has been demonstrated that several anti-*Babesia* drugs, such as diminazene aceturate and atovaquone, which possibly inhibit mitochondrial respiratory activity and electron transport, are ineffective owing to drug resistance [23–25]. Therefore, it is necessary to understand the mechanism of resistance of these anti-*Babesia* drugs and develop new drugs.

Studies of mitochondrial genomes could provide new insights into the biological features, genetic evolution and classification of the causative agents, as well as providing data for designing anti-*Babesia* compounds. To date, sequencing of the mitochondrial genomes has been performed for *B. bigemina*, *Babesia bovis*, *B. caballi*, *B. canis*, *B. conradae*, *B. duncani*, *B. gibsoni*, *B. microti*, *B. orientalis*, *B. rodhaini*, *B. rossi*, *B. vogeli*, *Theileria equi* and *T. orientalis* [26–31]. However, there are limited data on the mitochondrial genomes of ovine *Babesia* spp. in China. Although the mitochondrial genomes of BmLT and BspXJ were sequenced by using Illumina sequencing technology, they have not been sequenced using the Sanger dideoxy chain-termination method for verification [32]. In this study, we sequenced six mitochondrial genomes of *Babesia* isolates that can infect small ruminants, and the assembled and annotated sequences were submitted to GenBank after comparison with those of other piroplasms. These data were used to clarify the phylogenetic relationships and classification of the babesiae and to determine novel molecular markers for identification of *Babesia* species. The present study provides valuable information for understanding mitogenome evolution among apicomplexan parasites, identifying diagnostic markers and screening drug targets.

## Methods

### Parasites and isolation of genomic DNA

The purified merozoites of six ovine *Babesia* isolates were provided by our laboratory [33]. Genomic DNA extraction, concentration measurement and quality evaluation were performed as previously described by Wang et al. [34]. All genomic DNA samples were kept at − 20 °C until use.

### Amplification and sequencing

The PCR primers were designed based on the reported genomic sequences of *B. bovis* and *B. bigemina* (GenBank: AB499088 and AB499085) (Additional file 1: Table S1). Mitochondrial genome fragments were amplified and cloned into the pGEM®-T Easy Vector (Promega, Beijing, China) for subsequent sequencing by Sangon Biotech Company.

### Genome assembly and annotation

Mitochondrial genomic fragments were assembled with CLC Genomics Workbench v.7.5.1. The mitogenome annotation was performed using DOGMA [35] and Artemis [36], followed by application of BLAST (http://blast.ncbi.nlm.nih.gov/Blast.cgi) to identify homologous proteins in apicomplexan parasites in the GenBank database. The tRNA genes were searched using tRNAscan-SE v.2.0 with the default search mode and other mitochondrial sequence sources [37]. The rRNA genes were annotated by searching previously reported rRNA sequences of *B. bovis*, *B. microti*, *B. orientalis* and *T. parva*. The genome comparisons were aligned using Mauve [38]. The sequences were deposited in the GenBank database under the accession numbers MK962313, MK962314 and MN605889-MN605892.

### Sequence alignment and amino acid conservation of the *cytb* gene

We referred to previous studies [18–22, 39–43] that used the mitochondrial cytochrome *bc*1 complex from *Saccharomyces cerevisiae* to determine the binding residues of the inhibitor (acting on the *bc*1 complex) by X-ray crystallography, spectroscopy and *cytb* sequence alignment and analysis. In a previous report, sequence alignments of the *cytb* amino acid residue from *S. cerevisiae*, *Plasmodium falciparum*, *Toxoplasma gondii*, *B. microti* and *Bos taurus* indicated that the drug binding residues of *cytb* are conserved. Therefore, we used MegAlign software to compare the *cytb* sequences of *S. cerevisiae*, *B. taurus*, *T. gondii*, *T. parva*, *B. microti*, *B. duncani*, *P. falciparum*, BspXJ/DH, BmLT/TZ and BmNX/HB to identify the main resistance-related mutations and drug-binding residues in the genus *Babesia*.

### Phylogenetic analysis

The concatenated sequences of *cytb* and cytochrome *c* oxidase subunit 1 (*cox*1) amino acid residues of 26 apicomplexan parasites (Table 1) were aligned using Clustal W implemented in MEGA v.6.06 (http://www.megasoftware.net/) software. Subsequently, a phylogenetic tree was constructed using MEGA v.6.06 with maximum likelihood (ML) or neighbor-joining (NJ) analysis based on the JTT with the Freqs model. *T. gondii* and *Plasmodium* spp. were used as the outgroup. Furthermore, phylogenetic analysis of the whole mitochondrial nucleotide sequence was conducted by the ML method using the Kimura 2-parameter nucleotide substitution model. Consensus trees were created after bootstrap analyses with 1000 replications.

**Table 1:** Mitochondrial genome sequences of apicomplexan parasites used in the present study

Taxon	Size/bp	A + T contents (%)	Form	Protein-encoding genes	Original host	Country of origin	GenBank ID
*Babesia* sp. Xinjiang (BspXJ-Sanger)^a1^	5767	70.87	Linear	*cox*1, *cox*3, *cytb*	Sheep	China	MK962313
*Babesia* sp. Dunhuang (BspDH)	5767	70.85	Linear	*cox*1, *cox*3, *cytb*	Sheep	China	MK962314
*Babesia motasi* Lintan (BmLT-Sanger)^b1^	5836	70.05	Linear	*cox*1, *cox*3, *cytb*	Sheep	China	MN605889
*Babesia motasi* Tianzhu (BmTZ)	5836	70.13	Linear	*cox*1, *cox*3, *cytb*	Sheep	China	MN605890
*Babesia motasi* Ningxian (BmNX)	5946	70.10	Linear	*cox*1, *cox*3, *cytb*	Sheep	China	MN605891
*Babesia motasi* Hebei (BmHB)	5946	70.06	Linear	*cox*1, *cox*3, *cytb*	Sheep	China	MN605892
*Babesia* sp. Xinjiang (BspXJ-Illumina)^a2^	6020	71.30	Linear	*cox*1, *cox*3, *cytb*	Sheep	China	KX698108
*Babesia motasi* Lintan (BmLT-Illumina)^b2^	5790	69.97	Linear	*cox*1, *cox*3, *cytb*	Sheep	China	KX698109
*Babesia bovis* T2Bo	6005	70.49	Linear	*cox*1, *cox*3, *cytb*	Bovines	USA	EU075182
*Babesia bovis*	5970	70.35	Linear	*cox*1, *cox*3, *cytb*	Bovines	Japan	AB499088
*Babesia bigemina*	5924	69.82	Linear	*cox*1, *cox*3, *cytb*	Bovines	Japan	AB499085
*Babesia orientalis*	5996	71.10	Linear	*cox*1, *cox*3, *cytb*	Water buffalo	China	KF218819
*Babesia caballi*	5847	70.43	Linear	*cox*1, *cox*3, *cytb*	Equines	USA	AB499086
*Babesia gibsoni*	5865	72.24	Linear	*cox*1, *cox*3, *cytb*	Canines	Japan	AB499087
*Babesia gibsoni* (WH58)	5865	72.21	Linear	*cox*1, *cox*3, *cytb*	Canines	China	KP666169
*Babesia canis canis*	5769	71.90	Linear	*cox*1, *cox*3, *cytb*	Canines	USA	KC207822
*Babesia canis vogeli*	5603	71.19	Linear	*cox*1, *cox*3, *cytb*	Canines	USA	KC207825
*Babesia canis rossi*	5838	71.24	Linear	*cox*1, *cox*3, *cytb*	Canines	USA	KC207823
*Babesia conradae*	5608	72.41	Linear	*cox*1, *cox*3, *cytb*	Canines	USA	KC207826
*Babesia microti*	11,109	64.36	Linear	*cox*1, *cox*3, *cytb*	Murines, humans	Germany	AB624353
*Babesia microti*	11,109	64.36	Linear	*cox*1, *cox*3, *cytb*	Murines, humans	Germany	AB624354
*Babesia microti*	11,109	64.36	Linear	*cox*1, *cox*3, *cytb*	Murines, humans	Germany	AB624355
*Babesia microti*	11,109	64.36	Linear	*cox*1, *cox*3, *cytb*	Murines, humans	Germany	AB624356
*Babesia rodhaini*	6929	70.69	Linear	*cox*1, *cox*3, *cytb*	Murines	Australia	AB624357
*Babesia rodhaini*	6929	70.69	Linear	*cox*1, *cox*3, *cytb*	Murines	Australia	AB624358
*Babesia rodhaini*	6929	70.69	Linear	*cox*1, *cox*3, *cytb*	Murines	Australia	AB624359
*Babesia rodhaini*	6929	70.69	Linear	*cox*1, *cox*3, *cytb*	Murines	Australia	AB624360
*Babesia duncani*	5893	68.15	Linear	*cox*1, *cox*3, *cytb*	Humans	USA	MH107387
*Babesia* sp. Coco	5612	71.22	Linear	*cox*1, *cox*3, *cytb*	Canines	USA	KC207824
*Cytauxzoon felis*	5945	70.83	Linear	*cox*1, *cox*3, *cytb*	Felines	USA	KC207821
*Theileria annulata*	5905	70.57	Linear	*cox*1, *cox*3, *cytb*	Bovines	Turkey	NT167255
*Theileria parva*	5924	70.07	Linear	*cox*1, *cox*3, *cytb*	Bovines	Kenya	AB499089
*Theileria parva*	5895	70.01	Linear	*cox*1, *cox*3, *cytb*	Bovines	Kenya	Z23263
*Theileria orientalis*	5957	70.72	Linear	*cox*1, *cox*3, *cytb*	Bovines	Japan	AB499090
*Theileria equi*	8246	70.94	Linear	*cox*1, *cox*3, *cytb*	Equines	USA	AB499091
*Plasmodium berghei*	5957	68.84	Linear	*cox*1, *cox*3, *cytb*	Murines	Turkey	AB558173
*Plasmodium malariae*	5968	70.12	Linear	*cox*1, *cox*3, *cytb*	Humans	Japan	AB489194
*Plasmodium knowlesi*	5957	69.48	Circular	*cox*1, *cox*3, *cytb*	Humans, macaques	Malaysia	AY722797
*Plasmodium vivax*	5947	69.50	Linear	*cox*1, *cox*3, *cytb*	Humans	Malaysia	DQ396549
*Plasmodium falciparum*	5967	68.38	Circular	*cox*1, *cox*3, *cytb*	Humans	India	KT119882
*Toxoplasma gondii*	2607	64.90	Linear	*cox*1, *cytb*	Cat, human	RH	JX473253

^a^a1 and a2 are the same sample, which was sequenced using Sanger and Illumina methods, respectively

^b^b1 and b2 are the same sample, which was sequenced using Sanger and Illumina methods, respectively

## Results

### Sequence analysis

**Fig. 1:**
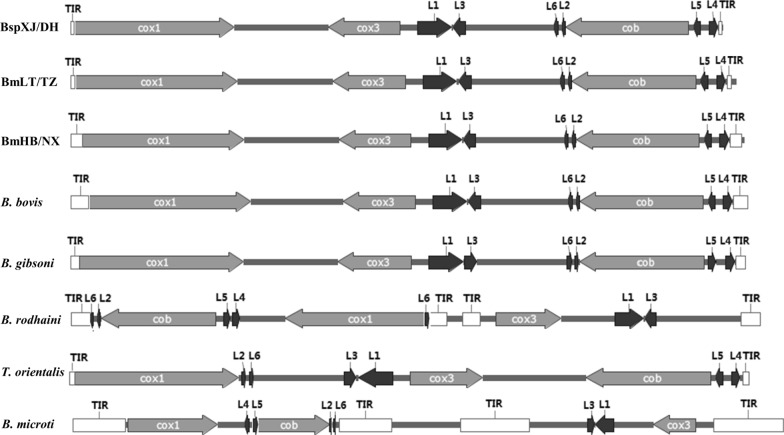
Comparison of the mitochondrial genomes of six ovine *Babesia* isolates and other apicomplexan parasites. The analysis was performed using SnapGene software and Adobe Photoshop. The different shades of gray represent different gene types. In detail, white represents TIR, 40% gray represents protein-encoding genes and 80% gray represents LSU

Sequence analysis revealed that the ovine *Babesia* mitochondrial genomes were linear DNA of 5767 to 5946 bp, with 70.05–70.87% A + T content (Table 1). Mitochondrial genomes of six ovine *Babesia* isolates contained three protein-encoding genes, *cox*1, cytochrome *c* oxidase subunit 3 (*cox*3), *cytb*, six large subunit rRNA genes (*LSU*) and two terminal inverted repeats (TIRs). The transcriptional direction of *cox*3, *LSU*3, *LSU*6, *LSU*2, *cytb* and *LSU*5 was from 3′ to 5′, whereas the direction of *cox*1, *LSU*1 and *LSU*4 was from 5′ to 3′ (Fig. 1, Additional file 2: Figure S1). The start codons of the *cox*3 and *cytb* genes of ovine *Babesia* were ATA and ATG, respectively. The initiation codon of the *cox*1 gene of BspXJ/DH was ATA, whereas that of *B. motasi* was ATG. Most of the protein-coding genes had TAA as a termination codon, followed by TGA (Table 2).

**Table 2:** Gene contents and initiation and termination codons of genes encoding the piroplasm mitochondrial genomes

Species	5′ TIR (bp)	*cox*1 (start, stop codons)	*cox*3 (start, stop codons)	*LSU*1 (bp)	*LSU*3 (bp)	*LSU*6 (bp)	*LSU*2 (bp)	*cytb* (start, stop codons)	*LSU*5 (bp)	*LSU*4 (bp)	3′ TIR (bp)
BspXJ/DH	25	1434 (ATA, TAA)	639 (ATA, TGA)	302	111	37	36	1092 (ATG, TAA)	69	82	25
BmLT/TZ	35	1434 (ATG, TAA)	639 (ATA, TGA)	297	111	37	36	1092 (ATG, TAA)	70	82	35
BmNX/HB	101	1434 (ATG, TAA)	639 (ATA, TGA)	297	111	37	36	1092 (ATG, TAA)	70	82	101
*B. gibsoni* (AB499087)	74	1434 (TTG, TAA)	642 (ATT, TAA)	306	111	43	35	1092 (TTA, TAA)	70	82	74
*B. bovis* (AB499088)	119	1434 (ATG, TAA)	639 (ATA, TAA)	302	111	38	35	1092 (ATG, TAA)	68	82	119
*B. duncani* (MH107387)	48	1302 (ATG, TAA)	588 (ATG, TAA)	298	111	44	34	1092 (ATG, TAA)	69	82	48
*B. bigemina* (AB499085)	65	1434 (ATG, TAA)	639 (ATA, TGA)	299	111	37	36	1092 (ATG, TAA)	70	82	65
*B. caballi* (AB499086)	62	1434 (ATA, TAA)	639 (ATA, TAA)	301	111	37	35	1092 (ATG, TAA)	68	82	62
*T. parva* (AB499089)	94	1440 (ATT, TAA)	642 (ATT, TAA)	301	111	38	38	1092 (ATG, TAG)	68	82	94
*T. orientalis* (AB499090)	47	1437 (ATA, TAA)	642 (ATT, TAA)	310	111	38	38	1092 (ATA, TAA)	69	82	47

### Comparison of mitochondrial genomes sequenced by Sanger and Illumina technology

The alignment of BspXJ and BmLT mitochondrial genomes sequenced using the Illumina method in a previous study [32] and the Sanger technique in this study was performed. The results showed that two sets of data from Sanger and Illumina have some differences in the size of the mitochondrial genome, the A + T contents, the number of LSUs, and the start and stop codons (Tables 1, 2, Additional file 3: Table S2) [32]. Furthermore, there were base differences at several positions between the two sets of sequences (Table 3).

**Table 3:** Comparison of *Babesia* sp. Xinjiang and *Babesia motasi* Lintan mitochondrial genomes were sequenced using Illumina (BspXJ-Illumina and BmLT-Illumina) and the Sanger method (BspXJ-Sanger and BmLT-Sanger)

Position^a^	1	2–195	179–185	218	5992–5993	5994–6052	5999–6044
*Babesia* sp. Xinjiang (BspXJ-Sanger, MK962313)		–…–			TT	–…–	
*Babesia* sp. Xinjiang (BspXJ-Illumina, KX698108)		AA…TT			AA	CT…TG	
*Babesia motasi* Lintan (BmLT-Sanger, MN605889)	A		GT…TT	–			AA…TT
*Babesia motasi* Lintan (BmLT-Illumina, KX698109)	T		–…–	G			–…–

Abbreviation: –, base deletion

^a^Position numbers given BspXJ (GenBank: MK962313)

### *cytb* gene sequence analysis

**Fig. 2:**
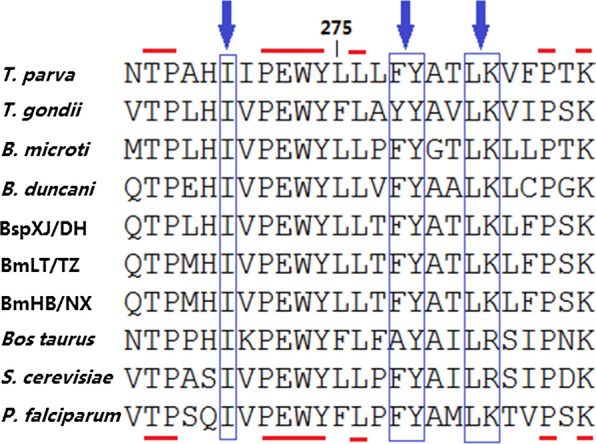
Amino acid sequence alignment of the conserved PEWY region of cytochrome *b* of six ovine *Babesia* isolates, *Theileria parva*, *Toxoplasma gondii*, *B. microti*, *Plasmodium falciparum*, *Saccharomyces cerevisiae*, *Bos taurus* and *B. duncani*. The alignment was constructed using MegAlign. The number 275 indicates to the residues at position Leu^275^ of *S. cerevisiae*. The residue at position 275 is a key determinant of the efficacy of ligand (atovaquone) binding to the *bc*1 complex. Arrows indicate amino acid positions altered in atovaquone-resistant parasites. Red horizontal lines indicate positions that are completely conserved in all species

The results of amino acid sequence alignment indicated that all *cytb* sequences contain the highly conserved PEWY motif. Leu^275^ of *S. cerevisiae* is a key determinant of the efficacy of atovaquone and myxothiazol binding to the *bc*1 complex. This position is occupied by a Leu in the *T. parva*, *B. microti*, *B. duncani*, BspXJ/DH, BmLT/TZ and BmHB/NX sequences, whereas a Phe is present in the *T. gondii*, bovine and *P. falciparum* sequences (Fig. 2). The inhibitors of Qi and Qo sites include atovaquone, stigmatellin, myxothiazol, endochin-like quinolone (ELQ), antimycin A and NQNO. The amino acid changes conferring atovaquone resistance in the yeast numbering system included five mutations (I269M, F278I/A, Y279C/S, L275F and L282V). The drug (target protein is Qo site of *bc*1 complex) binding residues of *cytb* of ovine *Babesia* included Met^128^, Gly^132^, Glu^259^, Leu^262^, Phe^265^ and Tyr^266^. Drug (target protein is Qi site of *bc*1 complex) binding residues of *cytb* of ovine *Babesia* included His^187^, Ser^191^ and Asp^214^ (Table 4).

**Table 4:** Main resistance-related mutations and drug binding residues of cytochrome *b* in *Saccharomyces cerevisiae*, *Plasmodium falciparum*, *B. microti*, *B. duncani* and six ovine *Babesia* isolates

Target protein	Drug	Amino acid changes conferring drug resistance in the yeast numbering system	*S. cerevisiae* residues	*P. falciparum* residues	*B. microti* residues	*B. duncani* residues	BspXJ/DH residues	BmLT/TZ residues	BmNX/HB residues
Qo site of the *bc*1 complex	Atovaquone	Ile^269^→Met, Phe^278^→Ile, Phe^278^→Ala, Tyr^279^→Cys, Tyr^279^→Ser, Leu^282^→Val, Leu^275^→Phe	Glu^272^, Leu^275^, Phe^278^, Tyr^279^	Glu^261^, Phe^267^, Tyr^268^	Glu^265^, Leu^268^, Phe^271^, Tyr^272^	Glu^257^, Leu^260^, Phe^263^, Tyr^264^	Glu^259^, Leu^262^, Phe^265^, Tyr^266^	Glu^259^, Leu^262^, Phe^265^, Tyr^266^	Glu^259^, Leu^262^, Phe^265^, Tyr^266^
	Stigmatellin	Leu^275^→Phe	Glu^272^	Glu^261^	Glu^265^	Glu^257^	Glu^259^	Glu^259^	Glu^259^
	Myxothiazol	Leu^275^→Phe	Leu^275^	–	Leu^268^	Leu^260^	Leu^262^	Leu^262^	Leu^262^
	ELQ-110	Met^139^, Gly^143^, Glu^272^	Met^139^, Gly^143^, Glu^272^	Met^133^, Gly^137^, Glu^261^	Met^134^, Gly^138^, Glu^265^	Met^126^, Gly^130^, Glu^257^	Met^128^, Gly^132^, Glu^259^	Met^128^, Gly^132^, Glu^259^	Met^128^, Gly^132^, Glu^259^
Qi site of the *bc*1 complex	Antimycin A	Asn^31^, Ser^34^, Gly^37^, Met^221^, Phe^225^, Lys^228^, Asp^229^	Ser^34^, His^202^, Lys^228^, Asp^229^	His^192^, Asp^218^	His^193^, Asp^220^	His^185^, Asp^212^	His^187^, Asp^214^	His^187^, Asp^214^	His^187^, Asp^214^
	ELQ-300	Ile^26^→Leu, Asp^229^	Ile^26^, Asp^229^	Ile^22^, Asp^218^	Asp^220^	Ile15, Asp^212^	Asp^214^	Asp^214^	Asp^214^
Qi and Qo sites of the *bc*1 complex	NQNO	Trp^30^, Asn^31^, Gly^33^, Gly^37^, His^204^, Ser^206^, Met^221^, Phe^225^	His^202^, Ser^206^, Asp^229^	His^192^, Ser^196^, Asp^218^	His^193^, Ser^197^, Asp^220^	His^185^, Ser^189^, Asp^212^	His^187^, Ser^191^, Asp^214^	His^187^, Ser^191^, Asp^214^	His^187^, Ser^191^, Asp^214^

### Phylogenetic analysis

**Fig. 3:**
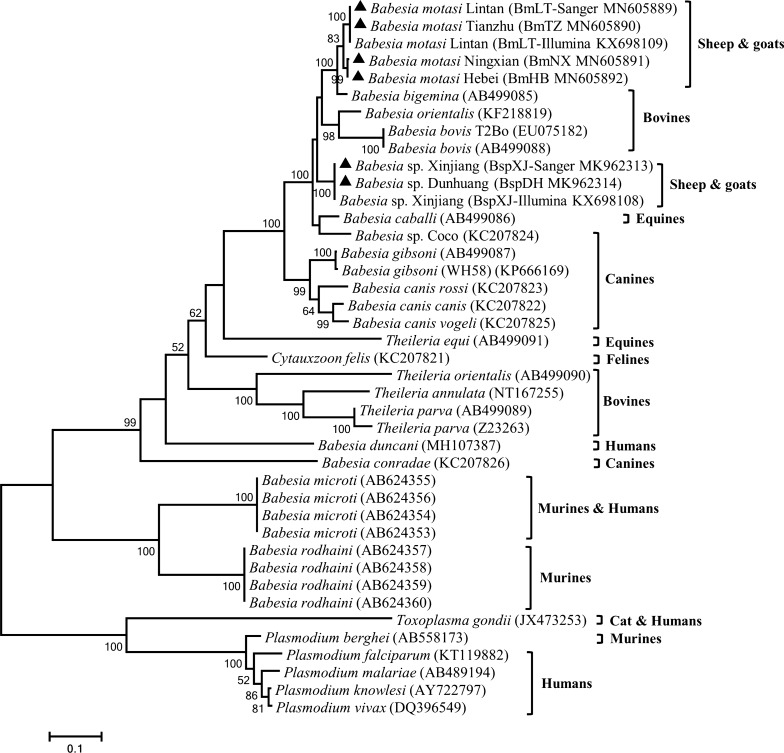
Phylogenetic relationships of *Babesia* infective to small ruminants in China and other apicomplexan parasites. Phylogeny was inferred with a maximum likelihood analysis of the amino acid sequences of the *cox*1 and *cytb* genes based on distances calculated with the JTT with the Freqs model. Bootstrap values > 50% from 1000 replicates are shown on the nodes. *Babesia* isolates examined in this study are indicated with triangles

Phylogenetic trees were constructed with the concatenated amino acid sequences of *cytb* and *cox*1 using the ML and NJ methods. The two approaches showed no significant changes in the topology. The piroplasms were divided into seven groups: (i) classical *Babesia* species that could infect ruminants, canines and equines; (ii) classical *Theileria* species that could infect bovines; (iii) *Theileria equi*; (iv) *Cytauxzoon felis*; (v) *Babesia duncani*; (vi) *Babesia conradae*; and (vii) *Babesia microti*/*Babesia rodhaini*. *Babesia* infective to ruminants were separated into four clades: *Babesia motasi*, *B. bigemina*, *B. orientalis*/*B. bovis* and *Babesia* sp. XJ/DH. Furthermore, *B. motasi* were further divided into two subclades: *B. motasi* LT/TZ and *B. motasi* NX/HB (Fig. 3). The phylogenetic tree using the whole mitochondrial nucleotide sequence was constructed by the ML method based on the Kimura 2-parameter model. The result was similar to that using concatenated amino acid sequences of *cytb* and *cox*1, with the exception of *T. orientalis*, which was located in the *B*. *conradae* clade (Additional file 4: Figure S2).

## Discussion

In the present study, we assembled and annotated the mitochondrial genomes of six ovine *Babesia* isolates and performed a mitochondrial genomic analysis with published mitochondrial genomes of apicomplexan parasites. The mitochondrial genomes of the six *Babesia* isolates infective to small ruminants are highly similar to those of most *Babesia* spp. with respect to genome size, high A + T content, genome form, and gene content. However, they are smaller than those of *B. microti*, *B. rodhaini* and *T. equi* and larger than that of *T. gondii* [28, 29, 44]. Consistent with the results from other apicomplexan parasites, tRNA genes were not found in the mitochondrial genomes. We speculate that they may be directly encoded by the nuclear genome. The order and transcriptional direction of three protein-encoding genes are the same in *B. bovis*, *B. bigemina* and *B. gibsoni* [28] but different from those in *B. microti*, *T. equi*, *T. orientalis* and *P. falciparum* [28, 29].

In the reported mitochondrial genomes of piroplasms, *B. microti* and *B. rodhaini* have a dual flip-flop inversion system, ranging from 184 to 1082 bp in length [29]. However, one pair of TIRs was found in the mitochondrial genomes of other *Babesia* spp. and *Theileria* spp., ranging from 25 to 1563 bp in length [26–28, 30, 32]. These findings indicated that the number and size of TIRs are one of the main causes of different mitochondrial genome sizes. We also found that different numbers of LSUs are responsible for the size of the mitochondrial genomes. TIRs are considered to play a crucial role in the replication and stabilization of linear mitochondrial genomes [45]. In the published mitochondrial genomes of apicomplexan parasites, the sequences of the coding genes and LSU are basically the same. Therefore, we speculated that differences in the lengths and sequences of TIRs may be responsible for divergence in the host-specific and *in vitro* culture characteristics of protozoans.

The difference between the Illumina and Sanger sequencing data is mainly caused by nucleotide substitutions, deletions and insertions, which result from the use of different sequencing techniques assembly and annotation software. The settings of the parameters are different, which is one of the reasons for the difference between the two datasets. The results of the Illumina sequencing method are more prone to errors than those of the Sanger method. The Sanger approach is more accurate and thus more appropriate for further studies of small genome sequencing.

Mitochondria are essential organelles and play an important role in energy metabolism, growth and development of apicomplexan protozoa [46]. Cytochrome *bc*1 is an integral membrane protein complex that is vital to cellular respiration. The highly conserved binding site of inhibitors in cytochrome *bc*1 is the molecular basis of the drug effect on yeast, fungi and parasites [43]. The drug binding residues in the *cytb* sequences of six ovine *Babesia* isolates are completely consistent. In the conserved PEWY region, Phe^278^ (yeast number of *cytb*) is present in most organisms, whereas *T. gondii* and *B. taurus* have Tyr and Ala, respectively, at this position. Studies have reported that the L275F mutation in yeast has no effect on enzyme activity, but the IC50 increased ten-fold [22]. Compared with the wild-type yeast, the Y279S mutant had a 40-fold increased IC50 (B1.7 mM) for atovaquone [22], and Y268S in the *P. falciparum* numbering system resulted in a 3000-fold loss of atovaquone sensitivity. In addition, the mutations M133I, L271V and Y268N of *P. berghei* confer resistance to atovaquone [43]. Therefore, we conclude that mutations in cytb are largely responsible for the efficacy of drugs (the target protein is the *bc*1 complex) in apicomplexan parasites.

Currently, atovaquone and ELQs have been reported for the treatment of human babesiosis and malaria by modifying the drug target through disruption of the cytochrome *bc*1 complex [19, 21, 22, 41, 43]. In 2019, a *B. motasi*-like parasite was detected in human blood in Korea [47], which suggests that *B. motasi* may be potentially zoonotic. Therefore, we should investigate the infection of *B. motasi* in humans in China and evaluate the zoonotic potential of *B. motasi* and the effect of inhibitors binding to the cytochrome *bc*1 complex. Our data showed that atovaquone, stigmatellin, myxothiazol, endochin-like quinolone (ELQ), antimycin A and NQNO drugs can be used in the treatment of babesiosis in the future. The molecular mechanism of the resistance of these drugs is the mutation of *cytb*, which suggests that a combined drug strategy is possible to avoid drug resistance during treatment of babesiosis.

In this study, the taxonomical relationships of *Babesia* isolates infective to sheep and goats are consistent with the reported phylogenetic analyses based on *cytb*, *cox*1, *cox*3, nuclear small subunit (*SSU*) and internal transcribed spacer (ITS) [5, 6, 8, 32, 48]. The ovine *Babesia* isolates are divided into two species: *Babesia* sp. and *B. motasi*. *Babesia motasi* further fell into two small clades, named BmLT/TZ and BmNX/HB. With the exception of *B. conradae*, piroplasm infective to the same host fell into one clade. These findings are consistent with the phylogenetic position of *B. gibsoni*, *B. duncani* and *B. orientalis* based on the amino acid sequences of *cox*1 and *cytb* [26, 27, 30].

## Conclusions

In conclusion, we reported the mitochondrial genome of six ovine *Babesia* isolates that infect small ruminants in China. The phylogeny based on the concatenated amino acid sequences indicated that there are two *Babesia* species (*Babesia* sp. and *B. motasi*) infective to sheep and goats in China and that the *B. motasi* isolates possibly belong to two subspecies (BmHB/NX and BmTZ/LT). The possible efficacy of the inhibitor (target protein is *bc*1 complex) should be evaluated on these six ovine *Babesia* isolates in future work. Further studies are needed to analyze the TIR and protein functions, which could provide new insights into the phylogenetic relationships, biology and therapy of *Babesia*.

## Supplementary information

**Additional file 1: Table S1.** Primers used for amplifying the mitochondrial genome of the six ovine *Babesia* isolates.

**Additional file 2: Figure S1.** Mitochondrial genome alignment of six ovine *Babesia* isolates with *B. bovis*, *B. bigemina*, *B. orientalis*, *Babesia* sp. Coco, *Cytauxzoon felis*, *T. parva*, *B. gibsoni*, *B. microti*, *B. rodhaini* and *P. falciparum*. The color blocks are linked by lines to similar blocks in genomes. The region of genomes covered by a color block is entirely collinear and homologous in the mitochondrial genomes.

**Additional file 3: Table S2.** Comparison of six ovine *Babesia* mitochondrial genomes.

**Additional file 4: Figure S2.** Phylogenetic tree of ovine *Babesia* isolates and other apicomplexan parasites. Phylogeny was created with a Maximum Likelihood method of mitochondrial nucleotide sequences using the Kimura 2-parameter nucleotide substitution model. The triangles represent the ovine *Babesia* obtained in our study.

## Data Availability

The datasets are included in the article and its additional files. The sequences were submitted to the GenBank database under the accession numbers MK962313, MK962314 and MN605889-MN605892.

## Abbreviations

DNA: Deoxyribonucleic acid
PCR: Polymerase chain reaction
cytb: Cytochrome *b*
*cox*1: Cytochrome *c* oxidase subunit 1
*cox*3: Cytochrome *c* oxidase subunit 3
*LSU*: Large subunit ribosomal RNA
TIR: Terminal inverted repeats
Qi: Quinone reduction
Qo: Quinol oxidation
ELQ: Endochin-like quinolone
SSU: Small subunit ribosomal RNA
ITS: Internal transcribed spacer
