# Supplementary material for: Insights into the phylogenetic relationships and drug targets of Babesia isolates infective to small ruminants from the mitochondrial genomes
Source: Parasit Vectors. 2020 Jul 29;13:378. doi: 10.1186/s13071-020-04250-8 (PMC7391622; doi:10.1186/s13071-020-04250-8)
Supplement: Supplementary file 1 — Additional file 1: Table S1. Primers used for amplifying the mitochondrial genome of the six ovine Babesia isolates. [file 13071_2020_4250_MOESM1_ESM.docx]

**Table S1** Primers used for amplifying mitochondrial genome of the six ovine *Babesia* isolates

| **Primers** | **Sequences** |
| --- | --- |
| mit1F1 | 5′-TTTATGATTCAAAGTTATAATTCG-3′ |
| mit1F2 | 5′-TTTCAGCAAATCATAAGATTATAGG-3′ |
| mit1B1 | 5′-ATATACTGTTTTATAATCCCATGCT-3′ |
| mit1B2 | 5′-ATTAGATACCCTGGGATATTCTGAT-3′ |
| mit1B3 | 5′-TAGCATTGTCTTATGTAGTTGTTCA-3′ |
| mit2F1 | 5′-CAAGCATAAGTATATAACATTGAAGG-3′ |
| mit2F2 | 5′-AGTGTGTTCAAAGTGGTTATTACG-3′ |
| mit2F3 | 5′-GCTATTAAGCCGATATAGAGTTTCA-3′ |
| mit2F4 | 5′-ATCAGAATATCCCAGGGTATCTAAT-3′ |
| mit2B1 | 5′-ATATACTAGATAGGGAACGAACTGC-3′ |
| BXJtir1R | 5′-GTGCCCAAACTAAACAACCTAACAA-3′ |
| BXJtir2R | 5′-GTCCAATAGACTTCAAAGCACCAAA-3′ |
| BXJtir1F | 5′-GGAAATCCAACACCATAACCACCTA-3′ |
| BXJtir2F | 5′-GCTGAGAAGCAGTTCGTTCCCTA-3′ |
| BLTtir-A | 5′-GCTGAGAAGCAGTTCGTTCCCTA-3′ |
| BLTtir-AS | 5′-TGATGAGCCCAAACTAGACAACC-3′ |
| BXJtir-A | 5′-CTGAGAAGCAGTTCGTTCCCTA-3′ |
| BXJtir-AS | 5′-CCATGTTTCGACTTCGTACTGAC-3′ |
