# Supplementary figures and images for: Insights into the phylogenetic relationships and drug targets of Babesia isolates infective to small ruminants from the mitochondrial genomes
Source: Parasit Vectors. 2020 Jul 29;13:378. doi: 10.1186/s13071-020-04250-8 (PMC7391622; doi:10.1186/s13071-020-04250-8)

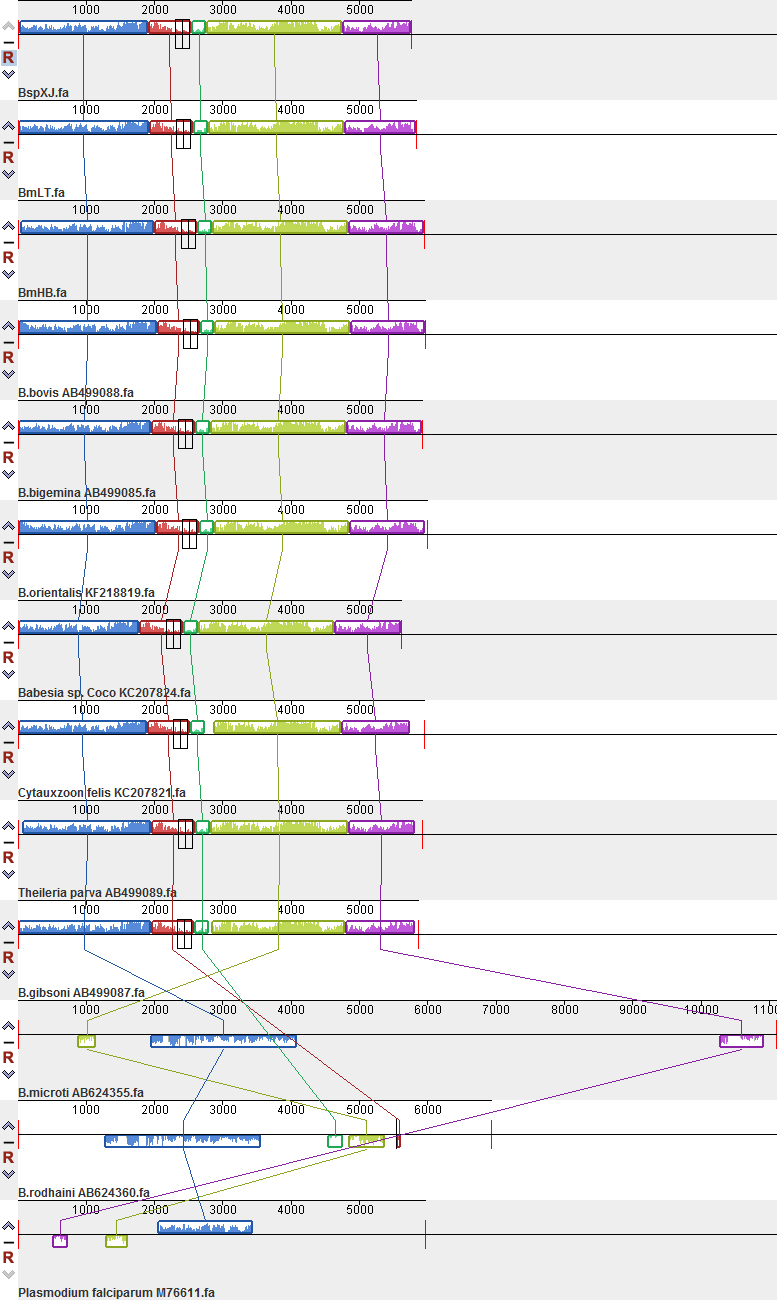

Supplement: Supplementary file 2 — Additional file 2: Figure S1. Mitochondrial genome alignment of six ovine Babesia isolates with B. bovis, B. bigemina, B. orientalis, Babesia sp. Coco, Cytauxzoon felis, T. parva, B. gibsoni, B. microti, B. rodhaini and P. falciparum. The color blocks are linked by lines to similar blocks in genomes. The region of genomes covered by a color block is entirely collinear and homologous in the mitochondrial genomes. [file 13071_2020_4250_MOESM2_ESM.png]

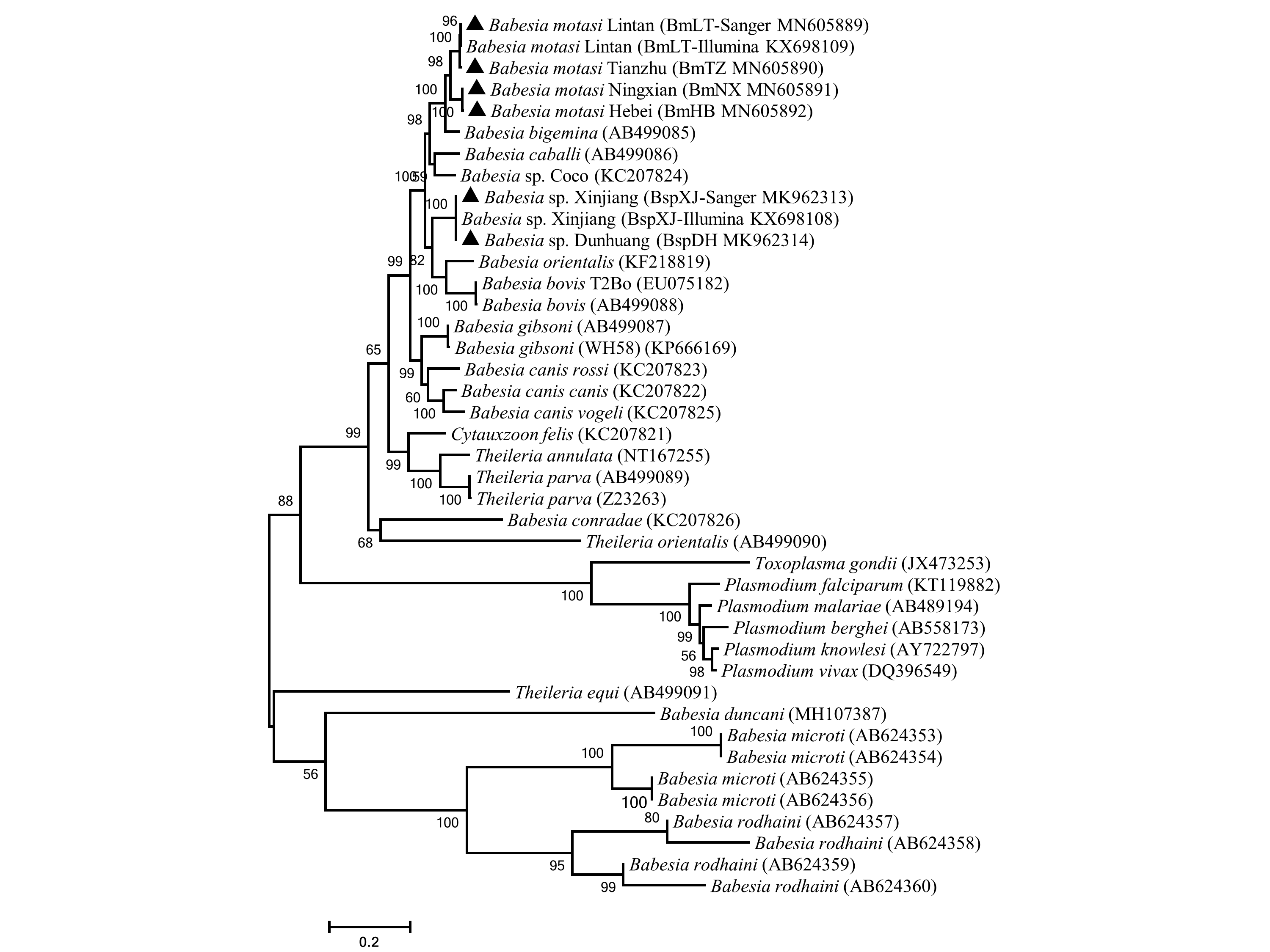

Supplement: Supplementary file 4 — Additional file 4: Figure S2. Phylogenetic tree of ovine Babesia isolates and other apicomplexan parasites. Phylogeny was created with a Maximum Likelihood method of mitochondrial nucleotide sequences using the Kimura 2-parameter nucleotide substitution model. The triangles represent the ovine Babesia obtained in our study. [file 13071_2020_4250_MOESM4_ESM.tif]
